# Nephropathia Epidemica Caused by Puumala Virus in Bank Voles, Scania, Southern Sweden

**DOI:** 10.3201/eid3004.231414

**Published:** 2024-04

**Authors:** Jiaxin Ling, Elin Economou Lundeberg, Anishia Wasberg, Inês R. Faria, Sanja Vucicevic, Bo Settergren, Åke Lundkvist

**Affiliations:** Uppsala University, Uppsala, Sweden (J. Ling, A. Wasberg, I.R. Faria, Å. Lundkvist);; Central Hospital of Kristianstad, Kristianstad, Sweden (E.E. Lundeberg, S. Vucicevic, B. Settergren)

**Keywords:** Puumala virus, viruses, Puumala orthohantavirus, zoonoses, nephropathia epidemica, etiology, phylogeny, rodents, bank voles, Sweden

## Abstract

In 2018, a local case of nephropathia epidemica was reported in Scania, southern Sweden, more than 500 km south of the previously known presence of human hantavirus infections in Sweden. Another case emerged in the same area in 2020. To investigate the zoonotic origin of those cases, we trapped rodents in Ballingslöv, Norra Sandby, and Sörby in southern Sweden during 2020‒2021. We found Puumala virus (PUUV) in lung tissues from 9 of 74 *Myodes glareolus* bank voles by screening tissues using a hantavirus pan–large segment reverse transcription PCR. Genetic analysis revealed that the PUUV strains were distinct from those found in northern Sweden and Denmark and belonged to the Finnish PUUV lineage. Our findings suggest an introduction of PUUV from Finland or Karelia, causing the human PUUV infections in Scania. This discovery emphasizes the need to understand the evolution, cross-species transmission, and disease outcomes of this newly found PUUV variant.

Viral zoonotic diseases give rise to most emerging or reemerging infectious diseases (*1*). Hantavirus infections are one of the most widespread rodent-borne viral zoonoses. The causative agents are orthohantaviruses (hantaviruses), which constitute a family of enveloped, single-stranded, negative-sense RNA viruses belonging to the family *Hantaviridae*, order *Bunyavirales.* The hantaviral virion comprises 3 RNA segments: the small (S) encodes for the nucleocapsid protein; the medium (M) segment for the glycoprotein precursor, which later will be cleaved into Gn and Gc; and the large (L) segment for the RNA-dependent RNA polymerase. 

The zoonotic transmission of hantaviruses to humans occurs primarily through indirect contact, such as inhalating aerosols from virus-contaminated rodent excreta or urine (*2*). The clinical symptoms of human hantavirus infections vary from asymptomatic to fatal hemorrhagic fever with renal syndrome (HFRS) or hantavirus pulmonary syndrome (HPS). The clinical manifestations are usually associated with the hantavirus species, carried by different rodents with distinctive ecologic habitats (*2*).

In Sweden, Puumala orthohantavirus (PUUV), carried by the bank vole (*Myodes glareolus*) (*3*,*4*), causes nephropathia epidemica (NE), which is a mild form of HFRS (*3*,*5*). However, PUUV may cause a similar level of severity or fatality to that of pathogenic Murinae-associated hantaviruses such as Hantaan virus and Seoul virus (*6*). Most of the clinical cases of NE are clustered in northern Sweden; incidence rate is 20 cases/100,000 population (*7*). Antibody prevalences of human PUUV infections, up to 16%, have been reported through serologic surveys (*8*). Although bank voles are present throughout the country, field-based ecologic studies of PUUV infections in rodents have mainly been performed in northern Sweden because of the positive correlation between bank vole density and the risk for human NE cases (*9*,*10*). Sweden has 2 distinct genetic lineages of PUUV circulating in the bank vole population: the North-Scandinavian (N-SCA) variant and the South-Scandinavian variant (S-SCA) (*3*) (Figure 1). However, increasing data indicate an expansion of the distribution of hantaviruses in rodents (*4*,*11*). Several serologic studies have revealed that PUUV-infected rodents have already reached far south of the traditional endemic areas of PUUV (e.g., in the Uppsala and Stockholm areas) (*4*,*11*).

**Figure 1:**
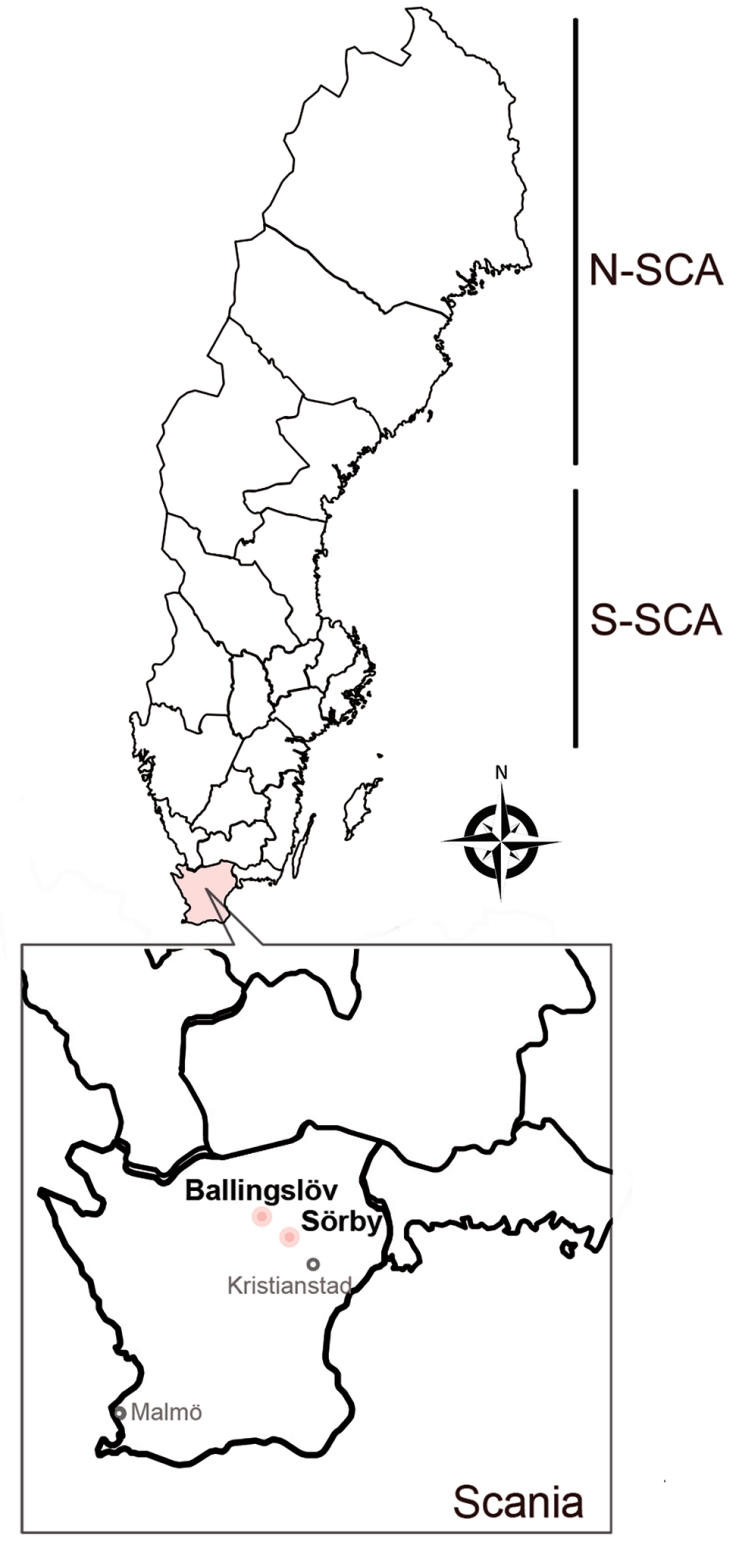
Rodent sampling sites for study of nephropathia epidemica caused by PUUV in *Myodes glareolus* bank voles in Scania, Sweden. New PUUV strains have been found in fields belonging to a patient with nephropathia epidemica in Scania. PUUV in Sweden belongs to the N-SCA lineage of PUUV, carried by bank voles of the Ural phylogroup, and S-SCA lineage of PUUV, carried by bank voles of the Carpathian phylogroup. Scania PUUV belongs to the Finland lineage of PUUV, carried by bank voles of the Carpathian phylogroup. N-SCA, North-Scandinavian; S-SCA, South-Scandinavian.

In southern Sweden, reported NE cases were travel-related until 2018, when a locally infected NE case was diagnosed in Scania, the southernmost region of Sweden (*12*). Another local NE case was diagnosed in the same geographic area in 2020. To determine the zoonotic sources of those human cases, we collected rodents close to the first patient’s home to characterize potential hantaviruses circulating in southern Sweden. 

To capture wild rodents for this study, we attained the required permits, including approval from the Malmö/Lund Ethics Committee on Animal Testing (reference 5.8.18–02281/2020). The Department of Infectious Diseases at the Central Hospital of Kristianstad was granted permission to handle laboratory animals from the Swedish Board of Agriculture (reference 5.2.18–14256/2019). The Swedish Environmental Protection Agency granted a hunting permit (reference NV-02812–20).

## Methods

### Sampling 

We collected of rodents during September 2020, May 2021, and September 2021 at 3 geographic sites in northeastern Scania: Ballingslöv (56°22′N, 13°87′E), Norra Sandby (56°20′N, 13°93′E), and Sörby (56°15′N, 13°98′E) (Figure 1). We placed the traps at all 3 locations but captured no rodents in Norra Sandby. We used mouse snap-traps and Supercat vole traps (Swissinno, https://www.swissinno.com) with dried apples and plums for bait. We placed traps during the day and collected trapped rodents the next morning. We then placed rodents in a −25°C freezer within 2 hours of collection. We then shipped the specimens in dry ice by express postal service to the Zoonosis Science Centre (ZSC) in Uppsala, where they were stored at −80°C until analysis.

### RT-PCR Screening

We dissected the rodents and harvested lung tissues for further molecular analyses. We extracted total RNA from rodent lung tissues using the QIAGEN RNeasy mini kit (QIAGEN, https://www.qiagen.com), followed by a hantavirus pan-L reverse transcription PCR (*13*). We further confirmed 9 PCR-positive samples using a PCR targeting the hantavirus S segment (*14*). We confirmed all 9 samples by partial sequences of both the PUUV S and L segments after Sanger sequencing by Macrogen Europe BV (https://dna.macrogen.com).

### Amplification of Host Cytochrome b Gene

We extracted total DNA from 14 rodent lung tissues using a tissue and blood DNA extraction kit (QIAGEN), then sequencing the mitochondrial cytochrome b (*cytB*) gene (*15*). We used the sequences for inferring the phylogeny of *M. glareolus* in Fennoscandia.

### RNA Sequencing

We sent 4 RNA samples to Novogene UK (Novogene Lab, https://novogene.com) for sequencing on the basis of the initial sequencing results. After ribosomal RNA depletion, the libraries were sequenced using paired-end sequencing with 150 bp per read and ≈50 million reads by the Illumina NovaSeq 6000 sequencing platform (https://www.illumina.com). A similar data analysis pipeline was described in a previous study (*16*). In brief, the raw clean reads were quality trimmed using Trimmomatic version 0.36 (https://github.com/timflutre/trimmomatic) and then mapped to the PUUV reference sequences from GenBank (S segment, NC_005224; M segment, NC_005223; L segment, NC_005225) and a mitochondrion complete genome of *M. glareolus* (GenBank accession no. MN122864) downloaded from National Center for Biotechnology Information RefSeq database using default settings for Geneious Prime version 2019.2.1 (https://www.geneious.com).

### Phylogenetic Analyses

To analyze the phylogenetic relationship of the novel viral and host sequences in comparison to other PUUV strains and rodent *cytB* sequences, we constructed phylogenetic trees using MrBayes version 3.2.6 (*17*). The reference strains and sequence information were based on earlier studies (*3*,*18*–*20*). We used MAFFT with default settings (*21*) to align global partial L-segment sequences and partial S-segment sequences; full length of S-, M- and L-segment sequence; and *cytB* sequences. We then used Aliview (*22*) for manual refinement. We determined best-fit model by jModeltest version 3.7 (*23*); we used general time-reversible plus frequencies plus invariate plus 4 matrix gamma model in all phylogenetic analyses. We used a MrBayes block for the computations with the setting including 5 million Bayesian Monte Carlo Markov chain (MCMC) generations sampling every 5,000 generations and obtained convergence (average deviation <0.01) with a 25% burn-in. We visualized all the tree results in FigTree version 1.43 (http://tree.bio.ed.ac.uk/software/Figtree) and edited them in Affinity Designer Pantone LL, 2019 (https://affinity.serif.com). We performed all computations using the UPPMAX computational cluster (https://www.uppmax.uu.se). We deposited all sequences generated in this study into GenBank (accession nos. OR602445‒8, OR607515‒23, OR581027‒41, and OR573657‒59).

## Results

### PUUV Variants in Scania

We collected a total of 74 rodent-like samples during 2020–2021 in the areas of the first NE patient in Scania. Morphologic identification during the dissection revealed 48 bank voles (*M. glareolus*), 25 *Apodemus* spp. mice, and 1 *Sorex* spp. shrew. After screening the lung tissues of all 74 animals, we found 9 bank voles positive by the hantavirus pan-L RT-PCR (Appendix Figure 1). We further confirmed all 9 samples for hantavirus infection by sequencing, followed by phylogenetic analyses based on global partial L-segment sequences (Appendix Figure 2). We found positive samples in 2 different geographic sites, Ballingslöv and Sörby. The phylogeny of the partial L sequences suggested that PUUV in southern Sweden has a close genetic relationship to PUUV circulating in Pallasjärvi, Finland; however, posterior probability (PP) was not supportive (PP = 0.46, which is <0.95). This finding might be explained by the short length of sequences that were used for the analyses. We further confirmed all 9 samples that were positive by the pan-L PCR with a method based on the PUUV S-segment as described previously (*14*). As expected, all 9 samples were found positive; the sequence results confirmed partial sequences of the PUUV S segment. Phylogenetic analyses based on 290 bp of the partial S sequences were in line with the tree of the partial L sequences and suggested that PUUV in southern Sweden belongs to the Finnish genetic lineage (Figure 1).

### Genetic Characteristics of PUUV Variants in Scania

To obtain a higher resolution of the phylogenetic relationship between the newly found PUUV and previously known PUUV variants, we chose 4 samples, 2_Ballingslöv_2021, 3_Ballingslöv_2021, 23E_Sörby_2020, and 25R1_Sörby_2020, to represent all 9 positive samples for RNA sequencing. However, 1 sample (2_Ballingslöv_2021) was later excluded from analyses because of bad sequencing quality. After mapping using implemented Geneious RNA in Geneious Prime version 2019.2.1, we found that a total of 23,165/106,347,176 reads from 3_Ballingslöv_2021, a total of 33,899/127,526,834 reads from 23E_Sörby_2020, and a total of 4,618/87,573,296 from 25R1_Sörby_2020 can be mapped to reference genome PUUV Sotkamo. After assembling, we obtained 3 PUUV full-genome sequences.

Comparative analyses of the new PUUV strains in Scania showed that they were closely related; diversity was 1.1%–1.8% nt and 0.2%–0.7% aa for the S segment, 3.2%–7.7% nt and 3.2%–8.1% aa for the M segment, and 2.9%–6.2% nt and 3.0%–6.3% aa for the L segment. Comparison with other PUUV strains circulating in Fennoscandia showed that all our Scania PUUV strains were more closely related to the Finnish strains. For the S segment, PUUV in Scania shared 90.0%–91.9% nt and 99.3%–100% aa sequence identities with Finnish PUUV strains (Sotkamo, Konnevesi, and Pieksamaki), 79.6%–80.1% nt and 96.5%–97.5% aa with S-SCA strains, and 79.1% nt and 96% aa with an N-SCA strain (Umeå_Human). For the M segment, they shared 90.7%–92.1% nt and 97.3%–98.6% aa sequence identities with Finnish strains (Sotkamo, Konnevesi, and Pieksamaki), 81.1%–83.4% nt and 92.0%–93.8% aa with S-SCA strains, and 81.2% nt and 92.3% aa with an N-SCA strain (Umeå_Human). For the L segment, they shared 91.4% nt and 98.3%–99.2% aa sequence identities with Finnish strains (Sotkamo, Konnevesi, and Pieksamaki) and 82.0% nt and 93.8% aa with an N-SCA strain (Umeå_Human).

We detected specific amino acid signatures in all 3 PUUV strains in Scania; they were R64K in the N protein and K798R in the glycoprotein. Of interest, 10 aa from 259–268 in the glycoprotein, located in Gn ectodomains, are missing in the 2 strains from Sörby. Sequence analyses did not find any specific amino acid signatures in the RNA-dependent RNA polymerase for those 3 strains.

### Phylogenetics of PUUV in Scandinavia

**Figure 2:**
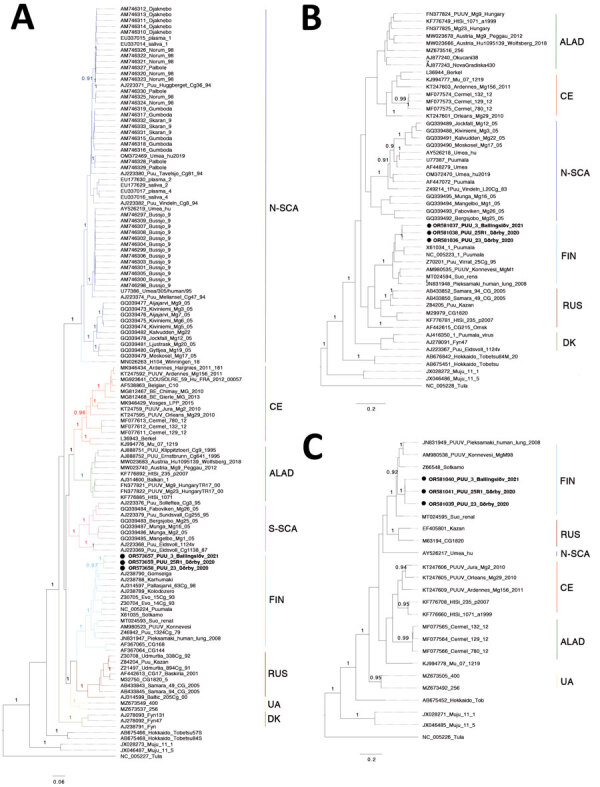
Bayesian phylogenetic trees based on full-length small (A), medium (B), and large (C) sequences from study of nephropathia epidemica caused by Puumala virus in *Myodes glareolus* bank voles in Scania, southern Sweden. Black circles and bold indicate sequences from this study. Puumala virus clusters: ALAD, Alpe-Adrian; CE, Central European; DA, Danish; FIN, Finnish; N-SCA, North-Scandinavian; RUS, Russian; S-SCA, South-Scandinavian; UA, Ukrainian.

We generated phylogenetic trees based on the full-length sequences of the S, M, and L segments (Appendix Figure 1). The topology of PUUV phylogenies was in agreement with other studies in which the current PUUV strains can be divided into well-supported clusters; in addition to N-SCA and S-SCA, Danish, Central European, Alpe-Adrian, Russian, Finnish, and Ukrainian lineages have been identified (*18*–*20**,**24*). In Sweden, the previously known lineages are S-SCA and N-SCA. S-SCA can be further divided into sublineages: the Mångelbo and Munga strains from Uppland; the Sollefteå, Bergsjöbo, and Fäboviken strains from Västernorrland; and the Eidosvoll strain from Norway. N-SCA can be further divided into the northern N-SCA lineage, including strains from Kiviniemi and Äijäjärvi, and the southern N-SCA lineage (*3*) (Figure 2, panel A). Surprisingly, the novel PUUV strains found in Scania did not belong to the S-SCA or the Danish lineages; instead, they clustered with the Finnish PUUV strains, suggesting an Eastern phylogroup as the common ancestor.

We further examined the mitochondrial DNA of the natural hosts that carry Scania PUUV. The sequencing results obtained by conventional PCR were not sufficient for reconstructing the phylogeny of *M*. *glareolus*. However, we recovered the host mitochondrial DNA from the RNA sequencing data; based on the mitochondrial *cytB* gene sequences, the phylogenetic tree suggested that all 4 bank voles that tested positive for PUUV belonged to the Carpathian clade, which is different from the bank voles in Finland of the Eastern phylogroup (Appendix Figure 3).

## Discussion

Our study discovered new PUUV variants in bank voles in Scania in southern Sweden, pinpointing the zoonotic source of local PUUV infection patient cases in southern Sweden. Unfortunately, we do not have access to any samples from those NE patients. However, by reviewing the diagnostic laboratory analysis of those NE cases along with our own results, we have established a genetic association between bank vole hosts in Scania and the local NE patients.

Our findings of Finnish-like PUUV variants in the bank vole population in southern Sweden raised the question of the origin of Scania PUUV. PUUV is the most common hantavirus in Europe, and its natural host, the bank vole, is widely distributed in large areas of Europe. Even though hantaviruses are thought to co-evolve with their hosts, PUUV is still absent in certain locations where bank voles are present (*25*). 

In Sweden, the diversity of hantaviruses has mainly been explored in northern rodents (*3*,*4*) because most of the clinical cases of NE are clustered in the north; a likely reason is the recolonization history of bank voles in Fennoscandia. After the last ice age ended in Fennoscandia, ≈10,000 years ago, the Ural phylogroup of bank voles migrated to the north of Fennoscandia and derived mtDNA from the northern red-backed vole (*Myodes rutilus*), whereas the southern bank voles of today originate from the Carpathian phylogroup, which had multiple colonizations to western and southern Fennoscandia (*26*). In contrast, there was 1 migration route from the northeast through Finland (*26*). Our phylogeographical results of bank voles in Scania based on mtDNA data are in agreement with all previous studies; that is, they belong to the Carpathian phylogroup and not the Eastern phylogroups. Nevertheless, PUUV in bank voles in Scania belongs to the Finnish PUUV lineage; a short branch in that clade implies a recent introduction of Finnish PUUV into Sweden.

Our current data cannot resolve the mystery of this recent introduction of PUUV in southern Sweden. One possible explanation might be that the virus, carried by bank voles, was transported from Finland or Russian Karelia (or other regions where the Finnish PUUV lineage is circulating) to Sweden and then established in bank voles there. The bank voles of the Eastern phylogroup carrying Finnish PUUV may have arrived in southern Sweden and spread the virus to Swedish bank voles but might not have been able to establish themselves as a unique phylogroup. Other less likely explanations might be an introduction via predatory birds or a reverse zoonotic transmission from humans to rodents. However, such possibilities are unlikely; only anecdotal reports on hantavirus-infected birds are available, and hantavirus transmission is known to be almost exclusively epizootic; just 1 human–human transmission has been reported, caused by Andes virus (ANDV) (*27*). Climate change or anthropic factors may have driven such cross-phylogroup transmission process, similar to what we have seen for the current distribution of Seoul hantavirus in Europe and worldwide (*28*). 

We have found PUUV in the neighborhood of 1 patient in the Sörby area, which indicates further dissemination of this PUUV variant in the local bank vole population. Our sequence analysis showed that 10 amino acids are missing in the glycoprotein of the 2 PUUV strains discovered in Sörby, which could be associated with the infectivity of PUUV. Our finding of 9 PUUV-positive samples from 48 captured bank voles suggests the prevalence of this recently introduced PUUV will probably not be restricted only to this patient’s fields; wider geographic distribution seems highly possible in Scania, given that the other NE patient was from northeastern Scania. However, our trapping focused on the first NE patient’s neighborhood; an ethical permit allowed us to capture <100 rodents, which may at least partially explain the high prevalence. However, our results indicate that more studies on the surveillance of rodents in southern Sweden will contribute to the understanding of the evolutionary mechanisms of virulence and transmission for PUUV variants in rodents, which requires more rodent samplings at various ecologic sites in Scania in the future. Furthermore, investigation into whether the Scania PUUV variant is more or less infective than the N-SCA and S-SCA variants in bank voles is warranted.

Novel PUUV strains in a new geographic area might have a substantial effect on human health. Since 2018, reported NE cases have not been increasing in Scania. However, the PUUV Pieksamaki strain, the closest relative of PUUV in Scania, has already caused 1 fatal case in Finland (*19*). In Finland, the incidence of diagnosed PUUV infections is ≈31–39 cases/100,000 population, and the fatality rate is ≈0.08%–0.4% (*29*). As of January 2024, only 2 NE cases reported from Scania were known to have been local infections. The patients’ symptoms included classic HFRS symptoms, such as fever, general malaise, nosebleed, and renal insufficiency, and laboratory results indicated thrombocytopenia and a moderately increased C-reactive protein. Those patients have fully recovered from their PUUV infections. We now need to understand the prevalence and pathogenicity of these new Scania PUUV strains in humans and their potential differences in virulence, compared with PUUV in N-SCA and in Finland. Future studies should isolate this new PUUV variant, and conduct larger serologic mapping surveys of rodents and febrile patients in Scania.

In conclusion, our study attempted to discover the zoonotic origin of NE cases in Scania, southern Sweden. Our identification of unique PUUV strains circulating in Scania provides critical insights into the pathogenic threats of emerging and reemerging viruses transmitted from rodents to humans. 

Appendix Additional information about nephropathia epidemica caused by Puumala virus in bank voles in Scania, southern Sweden.
