# Supplementary material for: Nephropathia Epidemica Caused by Puumala Virus in Bank Voles, Scania, Southern Sweden
Source: Emerg Infect Dis. 2024 Apr;30(4):732–7. doi: 10.3201/eid3004.231414 (PMC10977816; doi:10.3201/eid3004.231414)
Supplement: Appendix — Additional information about nephropathia epidemica caused by Puumala virus in bank voles in Scania, southern Sweden. [file 23-1414-Techapp-s1.pdf]

Article DOI: https://doi.org/10.3201/eid3004.231414

EID cannot ensure accessibility for supplementary materials supplied by authors. Readers who have difficulty accessing supplementary content should contact the authors for assistance.

Nephropathia Epidemica Caused by Puumala Virus in Bank Voles, Scania, Southern Sweden

Appendix

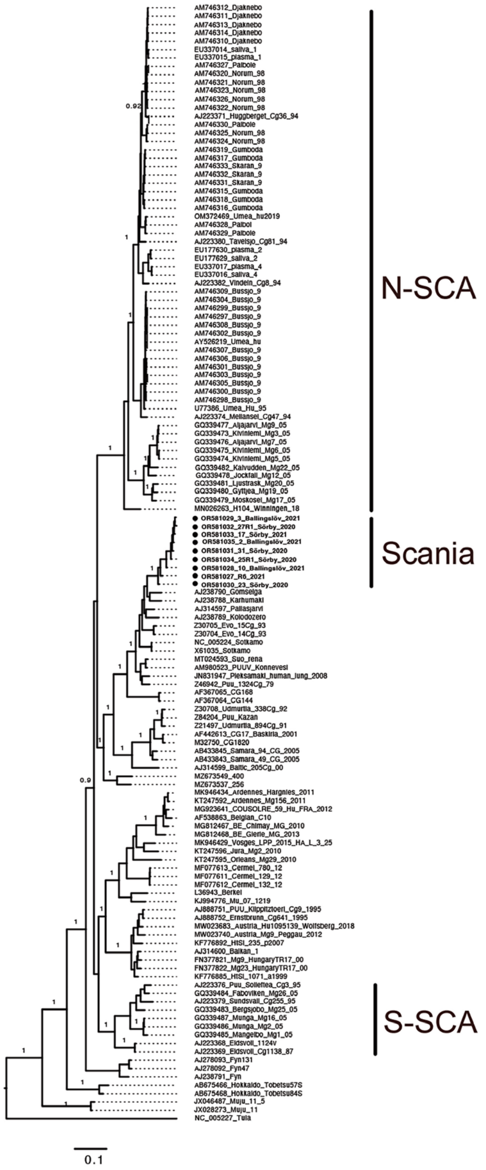

Appendix Figure 1. Phylogeographic distribution of PUUV and *Myodes glareolus* voles in Scania, Sweden. Bayesian phylogenetic tree is based on partial small sequences (292 nt) of PUUV. Dots indicate strains from this study. New PUUV strains have been found in fields belonging to a patient with nephropathia epidemica in Scania. PUUV in Sweden belongs to the N-SCA lineage of PUUV carried by bank voles of the Ural phylogroup and S-SCA lineage of PUUV carried by bank voles of the Carpathian phylogroup. Scania PUUV belongs to the Finnish lineage of PUUV carried by bank voles of the Carpathian phylogroup. Scale bar indicates nucleotide substitutions per site. N-SCA, North Scandinavia; PUUV, Puumala virus; S-SCA, South Scandinavia.

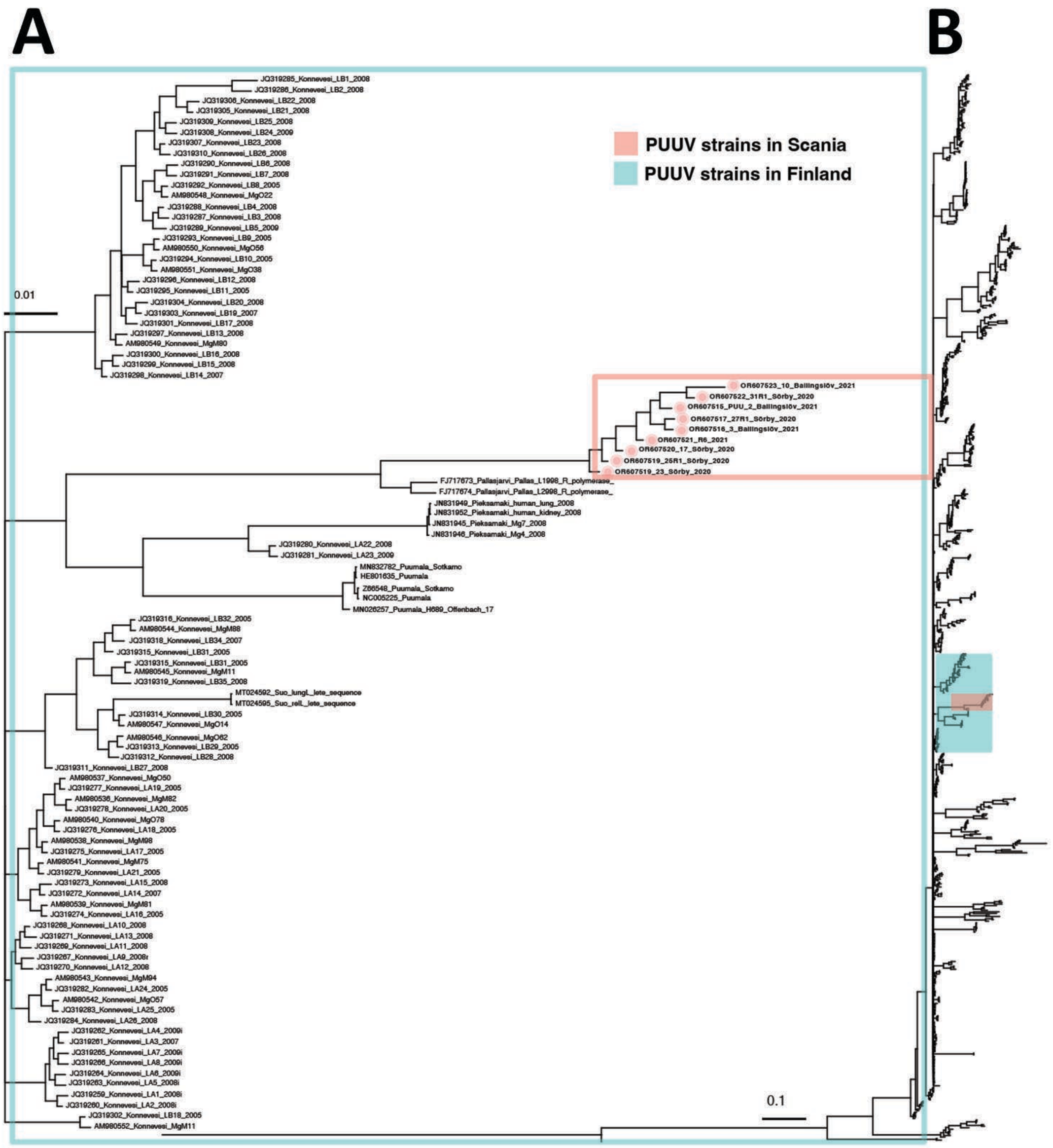

Appendix Figure 2. Bayesian phylogenetic trees based on partial large sequences. The sequences from this study have been highlighted in bold. A) A subset tree that is highlighted in the original tree. B) Pink color indicates PUUV strains in Scania, Sweden and light blue color indicates PUUV strains in Finland.

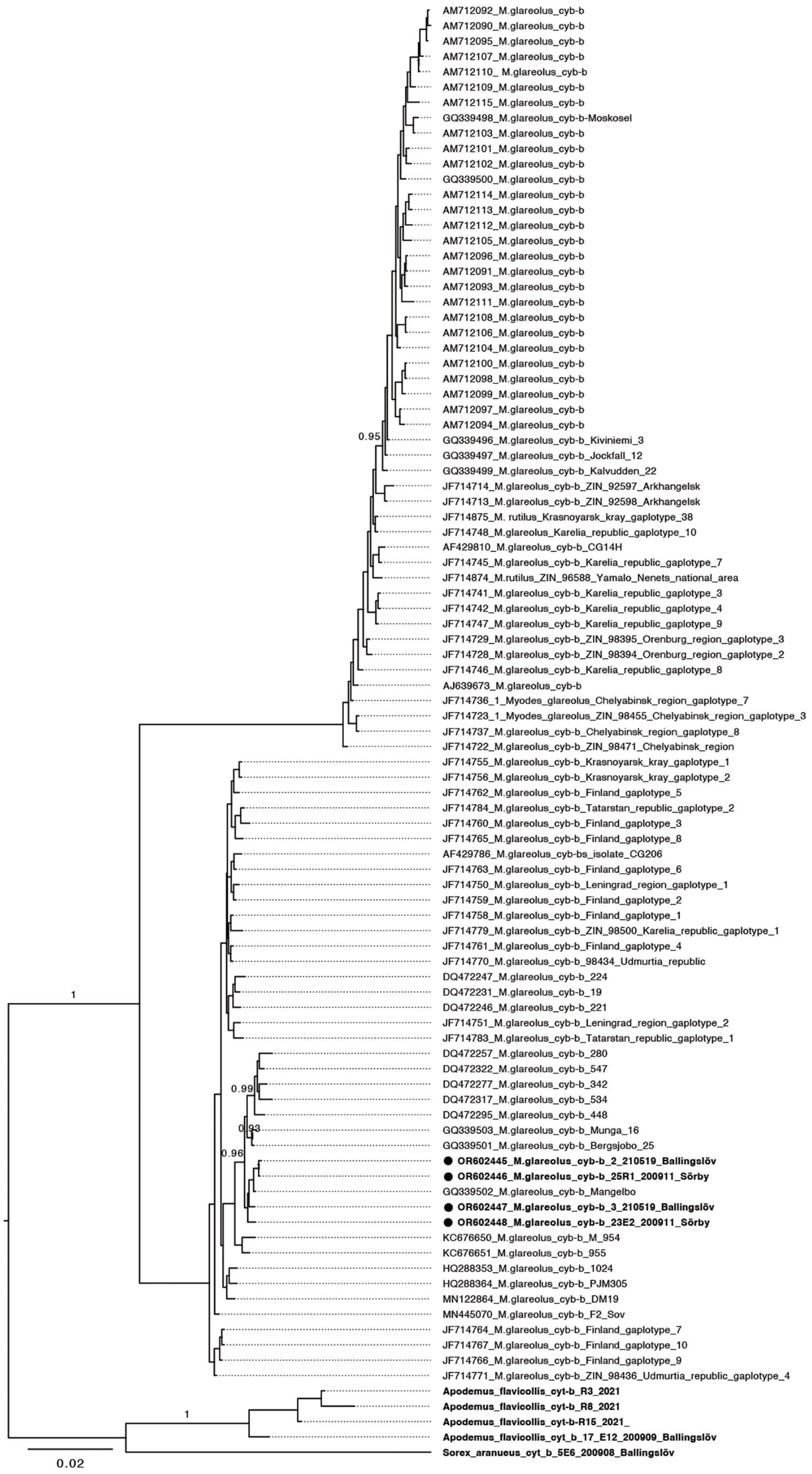

Appendix Figure 3. Phylogenetic tree of *M. glareolus* based on the partial sequence of the mitochondrial cytochrome b gene. The sequences from this study have been highlighted in bold. *M. glareolus* from Sörby and Ballingslöv fell into the same lineage as *M. glareolus* from Mangelbo, Munga, and Bergsjöbo (*3*).
